# Breaking and entering: predators invade the shelter of their prey and gain protection

**DOI:** 10.1007/s10493-015-9951-y

**Published:** 2015-07-19

**Authors:** Felipe Lemos, Ana Maria Guimarães Bernardo, Cleide Rosa Dias, Renato Almeida Sarmento, Angelo Pallini, Madelaine Venzon, Arne Janssen

**Affiliations:** IBED, Section Population Biology, University of Amsterdam, Science Park 904, 1098 XH Amsterdam, The Netherlands; Department of Entomology, Federal University of Viçosa, Viçosa, MG Brazil; Department of Plant Science, Federal University of Tocantins, Gurupi, TO Brazil; Agriculture and Livestock Research Enterprise of Minas Gerais (EPAMIG), Viçosa, MG Brazil

**Keywords:** Intraguild predation, Anti-predator behaviour, Ecological engineer, Shelter construction, Protection, Refuge, Prey preference, Oviposition site selection, Acari, Phytoseiids

## Abstract

Many herbivorous arthropods construct shelters on their host plant that offer protection against natural enemies. This has resulted in selection on natural enemies to enter these shelters, where they can feed on prey that are inaccessible for competing predators and parasitoids. The spider mite *Tetranychus evansi* produces a shelter consisting of a dense web that is impenetrable for most predators; the only known natural enemy that can penetrate the web and can forage efficiently on this pest is *Phytoseiulus longipes*. We show that this predator preferentially foraged and oviposited in the web of its prey. Moreover, intraguild predation on juveniles of these predators was significantly higher outside this web and in the less dense web of a closely related prey species (*T. urticae*) than inside the web of *T. evansi*. Although the production of shelters by herbivores may be profitable at first, their adapted natural enemies may reap the benefit in the end.

## Introduction

Many herbivorous arthropods construct shelters on their host plant, consisting of rolled or folded leaves and galls (Fukui 2001; Bailey et al. 2009) or webs that cover leaves or plants. These shelters offer protection against adverse abiotic conditions, natural enemies, competitors, and anchor the herbivores on the plants (Gerson 1985; Damman 1987; Hunter and Willmer 1989; Fukui 2001; Horita et al. 2004; Lemos et al. 2010; Sarmento et al. 2011b). This so-called ecosystem engineering (Jones et al. 1997) does not only affect the shelter-constructing herbivore and its interactions with its natural enemies, but can have profound effects on other organisms that use the shelter (Jones et al. 1997; Pallini et al. 1998; Fukui 2001; Magalhães et al. 2007). The use of shelters as refuge from natural enemies has in turn resulted in selection on predators and parasitoids to break and enter these shelters, and these natural enemies may secondarily also gain protection once entered (Roda et al. 2000).

Many phytophagous spider mites produce shelters consisting of silken webs (Saito 1985; Gerson 1985). Under these webs, colonies with high densities of mites feed on leaf parenchyma cells, thus causing significant damage to the plant. The webs are thought to protect spider mites against predation (Mori et al. 1999), and indeed, many predatory mites are hindered by the web (Sabelis and Bakker 1992). However, several predatory mite species are capable of entering the webs by breaking the strands (Montserrat et al. 2008; Shimoda et al. 2009). It has been hypothesized that the ability of predatory mites to move inside the web is correlated with the length of their dorsal setal pattern (Sabelis and Bakker 1992; Shimoda et al. 2009). These specialist predators often use the web as cues when foraging, restricting their searching to those plant parts that are covered with web (Hoy and Smilanick 1981; Sabelis 1981; Sabelis et al. 1984; Zhang et al. 2000; Horita et al. 2004), and preferentially ovipositing inside the web. In response, resting stages of some spider mite species reside outside the web, where the predation by such specialist predators is lower (Oku et al. 2003). Other organisms can also use spider mite web to their benefit. For example, thrips and predatory mites find protection from their predators inside the web (Pallini et al. 1998; Roda et al. 2000; Venzon et al. 2000).

A spider mite that constructs dense, chaotic webs is *Tetranychus urticae* (Saito 1985), which can feed and reproduce on over 1100 plant species (Migeon and Dorkeld 2015). Several species of predatory mites, mainly of the genus *Phytoseiulus* but also *Neoseiulus californicus*, can cope with the web of this species and successfully feed and reproduce on *T. urticae* (Bravenboer and Dosse 1962; De Moraes and McMurtry 1985; Friese and Gilstrap 1985; Oliveira et al. 2007). The closely related spider mite *Tetranychus evansi* is specialized on solanaceous plants (Ferragut and Escudero 1999). It is known to decrease defence levels in tomato plants (Sarmento et al. 2011a) and to produce extremely dense web (Ferragut and Escudero 1999), even denser than that of *T. urticae* (Sarmento et al. 2011b). The only predatory mite species known to effectively attack this spider mite is the predatory mite *P. longipes* (Badii et al. 1999; Furtado et al. 2007; da Silva et al. 2008); other species are hindered by the web of *T. evansi* (F. Lemos pers. obs.). Because of this unique capacity to enter the web of *T. evansi*, *P. longipes* could theoretically use this dense web as protection against intraguild predation (Holt and Polis 1997) by other predatory mites. To test this, we assessed the tendency of this predator to forage and oviposit inside the web of *T. evansi*, and measured the effect of the web on intraguild predation of young *P. longipes* by the closely related and co-occurring neotropical predatory mite *P. macropilis*.

## Materials and methods

### Plants and mite rearing

Tomato plants (*Solanum lycopersicum* var. Santa Clara I-5300) were weekly sown in a commercial plant substrate (Bioplant^®^, Bioplant Misturadora Agrícola, Nova Ponte, MG, Brazil) in a polystyrene tray (8 × 16 cells) in a greenhouse. Trays with seedlings were kept inside a cage covered with fine mesh to avoid contamination with other herbivores. After twenty days, seedlings were transplanted to 5 l pots containing the same substrate. The plants were ferti-irrigated weekly with a mixture of 50 g of N-P-K (20-05-20) and 100 g of simple superphosphate dissolved in 20 l of water. Pots were kept inside larger mesh-covered cages in a greenhouse.

A population of *T. evansi* was obtained in 2002 from a natural infestation on tomato plants in a greenhouse in Viçosa, MG, Brazil. A *T. urticae* population was started with individuals collected from naturally infested bean plants (*Phaseolus vulgaris*) in a greenhouse on the campus of the Federal University of Viçosa. Both spider mites were reared on tomato leaves that had their petioles inserted in a PVC tube filled with water to prevent desiccation of the leaves. Tubes with infested leaves were kept in PVC trays filled with detergent and water (1:25, v/v), which served to prevent mite escapes and invasion of mites and other non-flying arthropods. The rearing of each spider mite species was maintained in separate room (25 ± 3 °C, 70–90 % relative humidity) with controlled photoperiod (12:12 L:D).

The rearing of the predatory mite *P. longipes* was started with individuals that were kindly sent by Prof. Gilberto de Moraes from the University of São Paulo, Brazil, in 2006. *Phytoseiulus macropilis* was obtained in 2008 from a field population on common bean plants attacked by *T. urticae*. Both *P. longipes* and *P. macropilis* were cultured in similar rearing units as the spider mites, but were supplied with leaves infested by *T. evansi* or *T. urticae* respectively instead of clean leaves. Colonies of the two species were maintained in separate rearing rooms (25 ± 3 °C, 70–90 % relative humidity). All experiments were carried out in a rearing room (25 ± 3 °C, 70–90 % relative humidity) with controlled photoperiod (12:12 L:D).

### Effect of web on predation and oviposition by *Phytoseiulus longipes*

Leaf discs (2.4 cm diameter) were cut from the basal part of tomato leaflets and arranged on wet filter paper, positioned on wet foam inside a tray (12.5 × 7.5 × 2.5 cm) filled with water. Leaf discs were cut so that the midrib divided the leaf disc into two halves. A thin strip of wet cotton wool was placed along de midrib with the ends touching the wet filter paper. This connection with the water kept the cotton strip wet and impeded spider mites from crossing the cotton wool. Subsequently, each leaf disc half was infested with 60 *T. evansi* females and they were allowed to produce web and oviposit for 24 h. Subsequently, the females were killed with a thin insect needle or removed with a thin paint brush, depending on the treatment. We then immediately counted the number of spider mite eggs on each half disc and removed the cotton strip. Next, an entomological pin was inserted at the centre of the disc, drilling the leaf midrib. The pin was used as a basis to release the predatory mites. We took adult females of *P. longipes* of unknown age from the laboratory colonies, always selecting mites with an expanded opisthosoma, indicating they were reproducing. With a fine brush, one adult female was placed on the head of the pin. Predators were released within 3 h of removing the spider mites. The predators could walk down the pin and choose freely between the disc halves. After 24 h, the numbers of spider mite eggs left and *P. longipes* eggs laid on each half disc were recorded. Treatments were alternated between disc halves (left or right) to correct for unforeseen asymmetries.

Using this set-up, we first evaluated the preference of *P. longipes* for either *T. evansi* eggs without web or *T. evansi* eggs with web. We killed adult spider mites by piercing them with a fine entomological needle, taking care to minimize damage to the web. The web was subsequently removed from one of the disc halves using a thin brush. Each leaf disc contained over 600 spider mite eggs, of which the predators consumed on average <40. There were 24 replicates. Predation on the two leaf disc halves was compared with a generalized linear model (GLM) with treatment and disc half as factors and a quasi-Poisson error distribution. Predators always laid eggs on only one of the two disc halves. We therefore analysed the incidence of oviposition rather than the numbers of eggs on each disc half with a GLM with a binomial error distribution. In all tests, treatment, side of the disc (left or right) and their interaction were entered as factors. Non-significant interactions and factors were removed until a minimal adequate model was reached (Crawley 2007). All the statistical analyses were performed with R (R Development Core Team 2013).

Subsequently, we evaluated the oviposition behaviour of *P. longipes* on similar leaf discs as above, but with one half of the discs covered with web but without *T*. *evansi* eggs, and the other half containing eggs but no web. To obtain a leaf disc half without eggs but with web, we used virgin adult *T. evansi* of <1 day old, which produce very few eggs. These were obtained by collecting females in the last quiescent stage (teleochrysalids) from the laboratory colonies 1 day before the experiment. After maturing, they were allowed to produce web on one side of the leaf disc. Mated females from the colony were put on the other leaf disc half to oviposit and produce web. After 24 h, we killed the spider mites on both sides as above and pierced the few eggs laid by young virgin females. The web was removed from the side with mated females, as above. There were 11 replicates. Oviposition data were analysed as above.

### Prey preference

Following the same procedure as described above, we tested the preference of *P. longipes* females for *T. evansi* or *T. urticae*, because these two spider mite species produce web of different density. First we offered leaf discs with web and eggs of *T. urticae* on one side and web and eggs of *T. evansi* on the other side. As in the previous experiments, we killed the female spider mites after 24 h, preserving the web. We also offered similar leaf discs, but without web; females and web were removed from both sides using a fine brush. As in the previous experiments, one adult female of *P. longipes* was released on the head of a pin and predation and oviposition were assessed 24 h later. There were 15 replicates with web and 21 replicates without web. Data were analysed as above.

### Effect of spider mite web on intraguild predation

Here, we assessed whether spider mite web can offer protection against intraguild predation. The intraguild predator used was *P. macropilis*, a species that occurs on tomato plants, where it attacks *T. urticae*, but not *T. evansi* (Rosa et al. 2005). Leaf discs (2.4 cm diameter) were cut from tomato leaflets and arranged on wet cotton wool on a Petri dish. Fifty adult female spider mites, either *T. urticae* or *T. evansi*, were allowed to oviposit and produce web on entire leaf discs for 24 h. They were subsequently killed with a thin entomological needle as above. The web was removed from half of the discs as above, the other discs were completely covered with web, and all discs contained spider mite eggs. Subsequently, ten eggs of *P. longipes* (intraguild prey) were transferred from the laboratory colonies to each disc with a fine brush. They were aligned along the main vein, and were allowed to hatch for 3 days. The larvae emerging could easily enter the spider mite web when present. Subsequently, one adult female *P. macropilis* was transferred from a cohort of females of 9 days old since egg to each disc. The eggs of *P. macropilis* were carefully removed from the discs with a fine brush every day, taking care not to damage the web. Predation of *P. macropilis* on juveniles of *P. longipes* was evaluated after 72 h by counting the immature stages alive and the preyed upon. In total, there were four treatments: with eggs of either *T. urticae* or *T. evansi*, and either with or without web. Each treatment had 12 replicates, except for *T. evansi* with web, where one replicate was lost because the adult predator died. The proportions of juvenile *P. longipes* surviving intraguild predation were analysed with a GLM with a binomial error distribution with spider mite species and the presence/absence of web as factors. The number of eggs produced by the intraguild predator was analysed with a GLM with a Poisson error distribution with the same factors.

## Results

### Effect of web on predation and oviposition by *Phytoseiulus longipes*

**Fig. 1:**
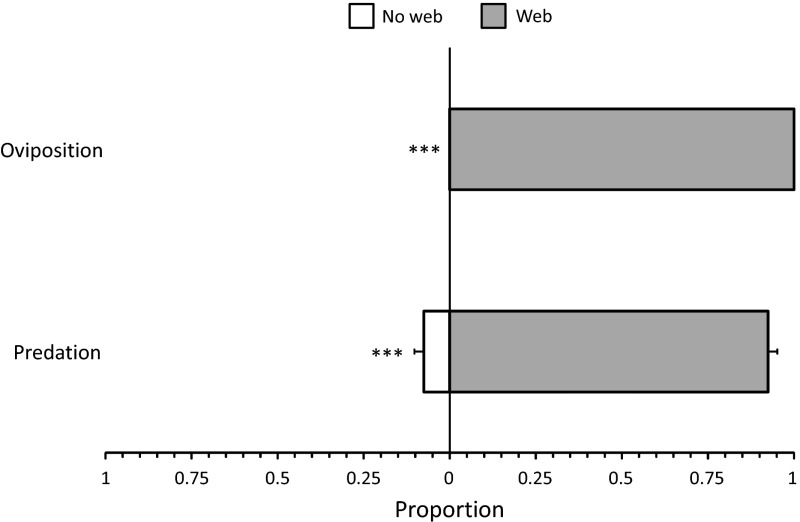
Preference of the predator *Phytoseiulus longipes* for web of its prey *Tetranychus evansi*. One half of a leaf disc contained eggs and web of *T. evansi* (*grey bars*), the other half contained eggs but no web (*white bars*). Predation is shown as the proportion of the total number of *T. evansi* eggs preyed on each disc half. Oviposition only occurred on the disc half with web. Asterisks indicate significant difference in proportion of prey eggs killed or predator eggs laid between leaf discs (GLM: ***, *P* < 0.001)

The predator *P. longipes* killed more eggs on the disc half with web of *T. evansi* than on the disc half without web (Fig. 1, GLM: F_1,46_ = 36.2, *P* < 0.0001) and oviposited exclusively on the disc half with web (Fig. 1, GLM: Deviance = 66.5, *d.f*. = 1,46, *P* < 0.0001). The oviposition rate of the predator was 3.1 (±0.19) eggs per disc per day.

When the predators were offered eggs of *T. evansi* on one disc half and web on the other, all predators oviposited exclusively in the web, except for one female that did not oviposit (GLM: Dev. = 33.2, *d.f*. = 1,20, *P* < 0.0001). Because the predators produce one egg at a time and need to feed in between ovipositions, this shows that the predators moved from the disc half with eggs where they fed to the disc half with web to oviposit. The mean oviposition rate of *P. longipes* was 2.2 (±0.4) eggs per female per day.

### Prey preference

**Fig. 2:**
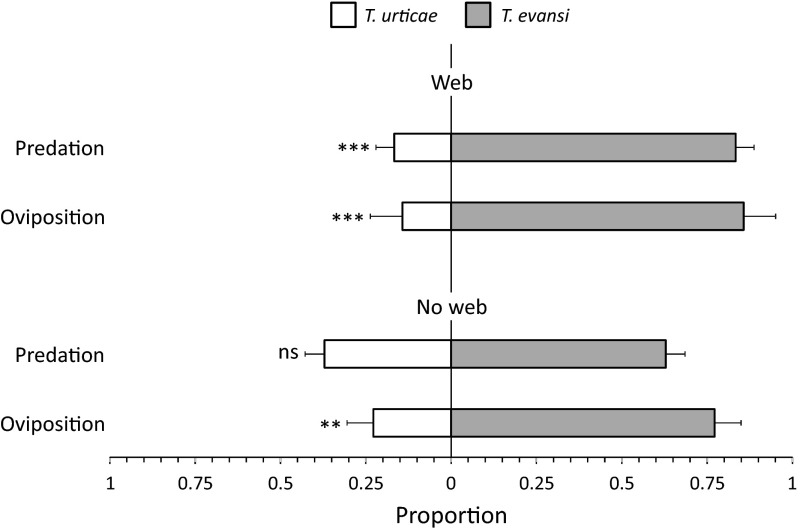
Preference of the predator *Phytoseiulus longipes* for web and eggs of two spider mite species. One half of a leaf disc contained eggs of *Tetranychus evansi* (*grey bars*), the other half contained eggs of *T. urticae* (*white bars*). *Top two bars* both disc halves contained web of the spider mites (*grey bars*: web of *T. evansi*, *white bars*: web of *T. urticae*). *Bottom two bars* the spider-mite web was removed from both disc halves. Oviposition is expressed as the average proportion of all predator eggs on either disc half. Predation is shown as the proportion of the total number of eggs preyed on each disc half. Asterisks indicate significant difference in proportion of prey eggs killed or predator eggs laid between leaf discs (GLM: **, *P* < 0.01 and ***, *P* < 0.001), *ns* no significant difference

In the presence of web produced by the prey, *P. longipes* killed more eggs of *T. evansi* than eggs of *T. urticae* (Fig. 2, top bar, GLM: F_1,28_ = 32.6, *P* < 0.0001), and oviposited more on the disc half with *T. evansi* (Fig. 2, 2nd bar from above, GLM: Dev = 14.7, *d.f.* = 1,28, *P* < 0.0001). The oviposition rate of the predator was 3.3 (±0.3) eggs per female per day.

Without web, *P. longipes* again killed more eggs of *T. evansi* than of *T. urticae*, but this difference was not significant (Fig. 2, 3rd bar from above, GLM: F_1,40_ = 1.02, *P* = 0.32). The oviposition rate was significantly higher on disc halves with *T. evansi* eggs (Fig. 2, lower bar, GLM: Dev. = 10.18, *d.f*. = 1,40, *P* = 0.0015). Overall, the oviposition rate of the predator was 2.3 (±0.3) eggs per female per day.

### Effect of spider mite web on intraguild predation

**Fig. 3:**
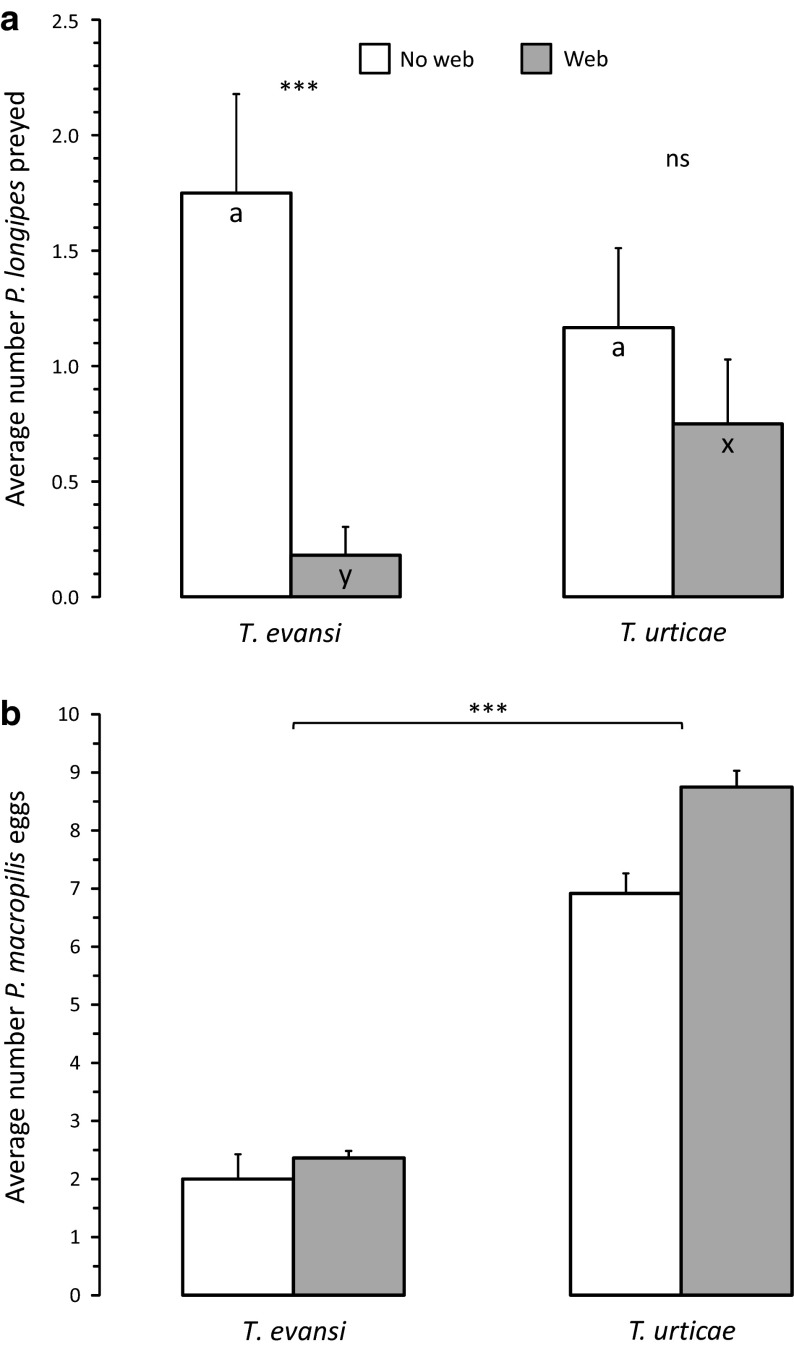
**a** Intraguild predation of juveniles of *Phytoseiulus longipes* in the presence of an intraguild predator, an adult female *P. macropilis*. Entire discs contained eggs of either *Tetranychus evansi* or *Tetranychus urticae* and were either covered with web (*grey bars*) of the same species or without web (*white bars*). Shown are average numbers (+ s.e.) of juveniles of *P. longipes* preyed during 3 days. There was a significant difference in intraguild predation on leaf discs with and without web of *T. evansi* (***, *P* < 0.001), and no such difference on leaf discs with and without web of *T. urticae* (ns). There was a significant interaction between the presence of web and the species that produced the web. Without web (*white bars*), there was no difference in intraguild predation on leaves with eggs of *T. evansi* or *T. urticae*, with web, there was a significant difference. **b** Average egg production (+ s.e.) by the predator *P. macropilis* during the intraguild predation experiment per 3 days. Predators oviposited significantly more on leaf discs with eggs of *T. urticae* than on discs with *T. evansi* eggs (***, *P* < 0.001)

Survival of juveniles of *P. longipes* in the presence of intraguild predators (i.e. *P. macropilis*) was significantly affected by the presence of web (GLM: Dev. = 13.3, *d.f.* = 1,44, *P* < 0.001) and by the interaction between the presence of web and prey species (Fig. 3a, GLM: Dev. = 6.12, *d.f.* = 1,43, *P* = 0.013). Intraguild predation was significantly lower in the presence of web of *T. evansi* than in the presence of web of *T. urticae*. Without web, there was no significant effect of the spider mite species on intraguild predation (Fig. 3a). This shows that the web of *T. evansi* offers better protection against intraguild predation than web of *T. urticae*. There was a significant effect of spider mite species on the oviposition rate of the intraguild predator (Fig. 3b: Dev. = 79.5, *d.f.* = 1,45, *P* < 0.001): the intraguild predator produced significantly more eggs on leaf discs with eggs of *T. urticae* than on discs with *T. evansi*. This shows that *T. urticae* is a significantly better prey for *P. macropilis* than *T. evansi* is.

## Discussion

The predatory mite *P. longipes* preferred foraging and ovipositing on leaf disc halves that were covered with spider mite web (Fig. 1), as was found for other predatory mites specialized in feeding on Tetranychidae (Hoy and Smilanick 1981; Sabelis 1981; Sabelis et al. 1984; Zhang et al. 2000; Horita et al. 2004). When eggs of *T. evansi* were offered without web, whereas the web on the other leaf disc half contained no food, the predators apparently commuted between the disc half with eggs to the disc half with web to oviposit. Because these predators need to feed to produce an egg, they commuted at least two times per day. This suggests that they have a strong preference to place their eggs inside spider mite web.

*Phytoseiulus longipes* preferred ovipositing on a disc half with eggs of *T. evansi* rather than on a disc half with *T. urticae*, even in the absence of spider-mite web (Fig. 2). This suggests that it can discriminate between the cues left behind by the two prey species. Without web, however, there was no significant preference for feeding on eggs of *T. evansi*, suggesting that the predators do not have a strong preference for the eggs of either of the two species.

Because the intraguild predator *P. macropilis* has difficulties to enter the web of *T. evansi*, we expected that juvenile *P. longipes* would be more protected from intraguild predation in the web of this prey species than in the web of *T. urticae*, and this is indeed what we found (Fig. 3a). Although we observed the adult predatory mites inside the web of both spider mite species, they did seem to move more sluggish inside the web of *T. evansi*, and this may have offered the juvenile *P. longipes* an opportunity to escape. The intraguild predators produced fewer eggs in the presence of *T. evansi* showing that the eggs of this predator species are less suitable food than eggs of *T. urticae*. In the presence of this inferior food, one would expect the intraguild predators to feed more on the intraguild prey than in the presence of superior food (i.e. eggs of *T. urticae*), and there was indeed such a trend in the absence of web, but it was not significant.

Theory predicts that intraguild predators and intraguild prey often cannot coexist (Holt and Polis 1997). Yet, in nature, they do coexist frequently enough to allow for recurrent observations of intraguild predation (Polis et al. 1989). Several mechanisms have been suggested that would decrease the strength of intraguild predation, thus promoting coexistence of species involved in intraguild predation (Mylius et al. 2001; Amarasekare 2007; Holt and Huxel 2007; Rosenheim 2007; Vance-Chalcraft et al. 2007). A meta-analysis has shown that habitat structure can decrease the strength of intraguild predation (Janssen et al. 2007), thus potentially promoting coexistence (but see Reichstein et al. 2013 for a counter-example). The spider mite web studied here is another example of a structure that decreases the strength of intraguild predation, but it remains to be investigated whether this promotes coexistence between the intraguild prey and intraguild predator studied here.

Our experiments show that predators that are capable of breaking and entering the refuge of their prey can thus gain protection from their enemies (Roda et al. 2000). In general, the production of shelters by spider mites and other herbivores must have offered protection at some point in evolutionary time. When natural enemies subsequently adapted to this defensive prey strategy, this benefit will have decreased. This is the more so because the predators and parasitoids could use the shelters for their own protection, resulting in lower mortality, and consequently in higher predation of the herbivores. Assuming that parasitoids are often more specialized than predators, this may offer an explanation for the observation that rates of parasitism of shelter-building herbivores are often higher than those of herbivores that do not construct shelters (Gentry and Dyer 2002; Abarca and Boege 2011; LoPresti and Morse 2013), although such interspecific comparisons should be treated with caution. However, there is no return for the herbivores: to stop building shelters would expose the herbivores to both generalist and specialist natural enemies.

In conclusion, we found that a predatory mite that is extremely specialized in penetrating the protective web of its prey does not only benefit from this because it gains access to an exclusive food source, but also because it can use the web for its own protection against intraguild predation.
